# Exploring offspring behaviour after prenatal COVID-19 vaccination in mice

**DOI:** 10.1038/s41398-026-04147-7

**Published:** 2026-06-15

**Authors:** Lilla Otrokocsi, Fruzsina Maácz, Pál Tod, Flóra Gölöncsér, Beáta Sperlágh

**Affiliations:** 1 Laboratory of Molecular Pharmacology, HUN-REN Institute of Experimental Medicine, Budapest, 1083 Hungary; 2 János Szentágothai School of Neurosciences, Semmelweis University School of PhD Studies, Budapest, 1085 Hungary

**Keywords:** Epigenetics and behaviour, Autism spectrum disorders

## Abstract

Maternal immunisation allows the transfer of protective antibodies to the offspring, reducing the risk of severe infection during early life. While vaccination during pregnancy is clinically recommended, its long-term impact on neurodevelopment remains under investigation. Autism spectrum disorder (ASD) is a neurodevelopmental condition characterised by social and behavioural alterations. Here, we evaluated whether prenatal exposure to the COVID-19 mRNA BNT162b2 vaccine or influenza vaccine affects general and autism-related behavioural outcomes in juvenile (4 weeks) and adult (8 weeks) mouse offspring. We also assessed maternal and fetal immune responses by measuring cytokines, soluble P2X7 receptor, BDNF, CRP, and lipid peroxidation in maternal plasma and fetal brain tissue. Prenatal COVID-19 vaccination elicited a moderate maternal cytokine response (increased IL-6 and KC) without overt fetal brain inflammation. Mild, age- and sex-dependent behavioural changes were observed, including anxiety-related parameters in the elevated plus maze and altered locomotion in the open field at 4–8 weeks, however, no consistent or robust ASD-like behavioural phenotype emerged across ages. Fetal P2X7 receptor levels were elevated, while fetal CRP, BDNF, and TBARS remained unchanged. In adult offspring, BDNF was selectively reduced in the prefrontal cortex, whereas hippocampal BDNF and P2X7 receptor levels in both regions were unaffected. Although embryonic P2X7 receptor levels were transiently elevated following COVID-19 vaccination, these changes were not accompanied by fetal inflammation or persistent adult purinergic alterations. Prenatal influenza vaccination induced mild, age-dependent behavioural alterations, such as increased circling in juveniles and reduced sociability in females, without detectable maternal or fetal inflammatory responses or changes in fetal neurotrophic markers. In contrast, maternal poly(I:C) administration provoked robust systemic inflammation and pronounced fetal brain effects, including elevated cytokines, CRP, P2X7 receptor, and region-specific microglial density, accompanied by persistent reductions in adult BDNF expression. Overall, within the limits of this experimental design, prenatal COVID-19 vaccination did not produce long-lasting ASD-like phenotypes or widespread neurodevelopmental alterations, while influenza vaccination had limited and transient effects. These findings are consistent with the absence of widespread or persistent neurodevelopmental disruption in this experimental model, although further studies—including multiple doses, gestational timepoints, and cellular-level analyses—are warranted to fully elucidate mechanisms and long-term outcomes.

## Introduction

The development of mRNA-based vaccines against COVID-19 represents a revolutionary breakthrough in modern vaccinology. Several studies have demonstrated their efficacy and safety in human populations, including pregnant women [1–3]. Recent findings have detected COVID-19 vaccine-derived mRNA in breast milk, suggesting systemic distribution beyond the injection site, including potential transfer to the placenta and fetus [4]. However, little is known about the long-term effects of their administration during pregnancy, particularly regarding neurobehavioural outcomes in offspring.

Maternal immune activation (MIA) may have a significant impact on neurodevelopmental processes in offspring, as demonstrated in a range of preclinical models. MIA is known to induce autism-like behaviours in animal models [5–7]. Prenatal immunostimulation leads to behavioural and neuroanatomical alterations relevant to neuropsychiatric disorders, including sensory gating deficits, high levels of repetitive behaviour, decreased social interactions, and altered cognitive function [7, 8]. In these MIA models, a relationship was found between behavioural changes and changes in maternal proinflammatory cytokine and chemokine profiles [9]. The P2X7 receptor has been proposed as a key mediator of inflammation, that can contribute to neurodevelopmental alterations [10, 11]. Clinical and experimental findings indicate that neuroinflammatory changes are accompanied by alterations in central and peripheral neurotrophin levels [12, 13]. Additionally, changes in brain-derived neurotrophic factor (BDNF) levels may indicate alterations in synaptic plasticity, which may correlate with neurodevelopmental anomalies in rodents [5, 14].

mRNA vaccines may affect fetal development via immune activation; however, the exact mechanisms and behavioural consequences remain unclear. Erdogan and colleagues [15] found altered BDNF levels in both male and female offspring after COVID-19 mRNA BNT162b2 vaccine-treated pregnant rats. Also, the behaviour of the male rats was characterized by a significant decrease in social interactions and motor performance. In humans, moderate developmental differences were observed in a retrospective population-level cohort study in infants born to mothers vaccinated against COVID-19 during pregnancy at 13–15 months [16], although potential neurodevelopmental effects at later ages remain to be investigated. However, other studies have reported an increased prevalence of developmental delays in infants prenatally exposed to SARS-COV-2 infection [17–19]. Together, these findings indicate that prenatal immune activation – whether infection- and vaccine-related - may have context-dependent neurodevelopmental consequences, underscoring the need for mechanistic preclinical investigations addressing long-term outcomes. Therefore, it is still an outstanding question how vaccines, in particular mRNA vaccines, administered during pregnancy may impact postnatal development of the nervous system in order to inform clinical guidance and tackle vaccine hesitancy.

This study aimed to analyse autism-related behavioural outcomes in offspring after treating pregnant mice with the COVID-19 mRNA BNT162b2 vaccine. In addition, to characterize the immune response after vaccination, we determined the levels of different cytokines, chemokines and soluble P2X7 receptor (P2X7R) in the plasma of pregnant animals on day 13.5 of gestation, 24 h after vaccine administration. E12.5 represents mid-gestation in mice, developmentally corresponding to the late first trimester in humans. This period is characterized by active cortical neurogenesis and neuronal migration and is particularly sensitive to immune-mediated disturbances. Accordingly, E12.5 has been widely employed in maternal immune activation models investigating long-term neurodevelopmental outcomes [20]. Additionally, brain-derived neurotrophic factor (BDNF) levels were assessed in the embryonic brain to better understand potential neurodevelopmental effects.

Our results may contribute to a deeper understanding of the safety of vaccination during pregnancy and provide insights into the relationship between the maternal immune response and postnatal neurodevelopmental patterns.

## Materials & methods

### Animals

Animals were housed under standard laboratory conditions (12:12 light-dark cycle, 23 ± 2 °C, 60 ± 10% humidity), with food and water available *ad libitum*. Environmental enrichment included cardboard bedding and tubes in each cage. To minimize suffering and reduce animal use, all procedures complied with the ARRIVE guidelines. Experimental protocols were approved by the local Animal Care Committee of IEM (Permission No: PE/EA/00149-4/2024). Male and female C57BL/6 mice were used (body weight: 12–18 g at 4 weeks, 20–25 g at 8 weeks). Mice (2–5 per cage) were housed with same-sex littermates on corncob bedding. Behavioral tests were performed during the dark phase, between 9:00 and 14:00, following a 30-minute habituation period in the experimental room.

### Experimental design

**Fig. 1:**
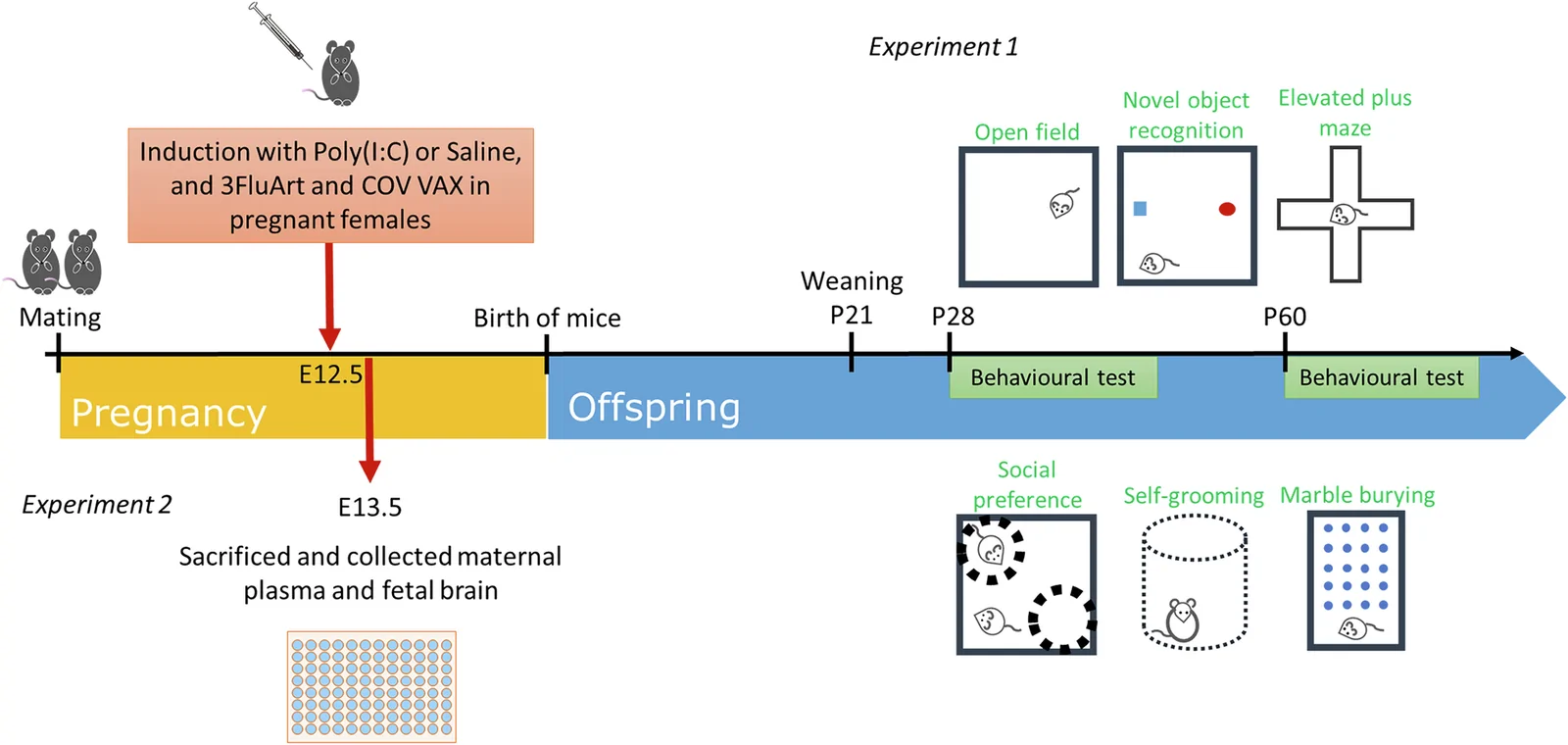
Experimental design. After mating and confirmation of pregnancy, poly(I:C), saline, 3FluArt influenza vaccine and COVID-19 mRNA vaccine were administered on prenatal day 12.5. Experiment 1: On postnatal day 21, the offspring animals were sexed and from days 28 and 60 behavioural test were performed. Experiment 2: On E13.5 pregnant dams were sacrificed and maternal blood and fetal brain tissue were collected.

Experimental design is shown in Fig. 1 in the Results section. Mating trios consisted of two females and one male per cage, housed together only for the duration of mating (designated as day 0). Primiparous dams (10–16 weeks old) were mated with sires aged 3–6 months. Sires were randomly assigned to females, regardless of the subsequent treatment groups (saline, poly(I:C), or vaccines). Pregnancy was confirmed 12 days post-mating, and pregnant females were randomly allocated to treatment groups.

On embryonic day (E)12.5, pregnant dams received a single intramuscular injection of the BNT162b2 mRNA vaccine at a dose of 10 µg per mouse in a volume of 100 µL. This dosing strategy was selected based on preclinical immunogenicity and regulatory data showing robust murine immune responses to BNT162b2 at microgram doses (0.2–5 µg per animal) in published preclinical studies and regulatory reports [21–25]. This dose corresponds to approximately one-tenth of the human dose when scaled to mouse body surface area, in accordance with non-clinical regulatory guidance for developmental toxicity testing. In prior murine studies, intramuscular administration of BNT162b2 at doses of 0.2, 1, and 5 µg induced robust neutralizing antibody responses in BALB/c mice (FDA, BNT162b2 Module 2.6.2. Pharmacology Written Summary). Furthermore, regulatory documents (FDA nonclinical summary) describe repeat-dose studies with higher doses in rodents without overt systemic toxicity (Pfizer-BioNTech COVID-19 Vaccine VRBPAC Briefing Document). This volume is widely used in murine vaccination studies and corresponds to the reduced-dose human formulation scaled to mouse body mass [26, 27]. The influenza vaccine contained 6 µg of inactivated purified surface antigen from each of the WHO-recommended strains [A/Victoria/4897/2022 (H1N1)pdm09-like, A/Darwin/9/2021 (H3N2)-like, and B/Austria/1359417/2021-like], adjuvanted with aluminum phosphate gel (max. 0.625 mg Al³⁺) [26, 28]. Maternal immune activation (MIA) was induced by intraperitoneal injection of poly(I:C) at 10 mg/kg body weight, in line with established mouse MIA models [29]. Control animals were administered 250 μL of sterile saline at the same time point.

Offspring were weaned at 4 weeks of age and housed in same-sex groups of 3–5 animals per cage. Behavioral testing was conducted on test-naïve male and female offspring at 4 and 8 weeks of age in the same sequence (open field, novel object recognition, elevated plus maze, social preference, self-grooming test, marble-burying test), by an experimenter blinded to the treatment.

In separate cohorts, pregnant dams were sacrificed 24 h after treatment (E13.5) to assess maternal immune responses and fetal brain development. Maternal blood samples were collected for cytokine bead array (CBA) analyses, and fetal brains were harvested for BDNF ELISA. Dams were deeply anesthetized with isoflurane (induction: 4–5%, maintenance: 1.5–2% in oxygen) using a precision vaporizer, and anesthesia depth was confirmed by the absence of paw withdrawal reflex. Terminal blood collection was performed via the vena cava using heparinized 25 G × 5/8″ needles, in compliance with Directive 2010/63/EU to ensure humane euthanasia. Embryos were collected on E13.5. At this developmental stage, embryos are not considered capable of conscious perception or pain; therefore, no additional anesthesia was required prior to brain tissue collection. All experiments were conducted on at least 2–3 independent litters.

### Drugs and treatments

The following drugs were used in our experiments: 3FluArt (Influenza A/B inactivated whole virion vaccine, ‘3FluArt’, Fluart Innovative Vaccines, 100 µl), BNT162b2/Comirnaty (Covid 19 mRNA vaccine, ‘COV VAX’, Pfizer-BioNTech, 100 µl), poly(I:C) (polyinosinic–polycytidylic acid potassium salt, ‘PIC’, InvivoGen, 10 mg/kg), and control was physiological saline solution (250 µl, ‘SAL’).

### Gel electrophoresis

The integrity of the vaccine was verified by electrophoretic separation on 2% agarose gel (Invitrogen, Waltham, MA, USA) in pH = 8.5 TAE buffer (composition: 242 g Tris Base, 57.1 ml glacial acetic acid, 100 ml 0.5 M EDTA). The wells were loaded with the total volume of 6 µl in the following allocation: 2 µl undiluted vaccine solution, 2 µl nuclease-free water (Fermentas, Waltham, MA, USA), 1 µl GelRed® Nucleic Acid Stain (Biotium Inc., Fremont, CA, USA) 1 µl 6x loading dye (Fermentas). For molecular weight ladder, 1 µl GeneRuler DNA Ladder Mix (Cat # SN0334), ready-to-use (ThermoFisher Scientific, Waltham, MA, USA) was used. Voltage was kept on constant 80 V (voltage source: Bio-Rad PowerPac Basic; Bio-Rad Laboratories, Inc., Hercules, CA, USA), and the separation lasted for 60 min. The agarose gel was visualised by ChemiDoc^TM^ MP Imaging System (Bio-Rad Laboratories Inc.) (Fig S6).

### Behaviour tests

#### Open field test

Locomotor activity was assessed in a square Plexiglas arena (40 × 40 cm). Mice were placed in the center of the arena and allowed to explore freely for 10 min. Movements were recorded and analyzed using the Noldus EthoVision XT 15.0 tracking system (RRID:SCR_000441, Noldus Information Technology, Wageningen, Netherlands). The total distance traveled (cm) was used as a measure of spontaneous locomotor activity.

#### Novel object recognition test

The test was performed in the same 40 × 40 cm black Plexiglas arena. During the habituation phase, mice were allowed to explore the empty arena for 10 min. In the familiarization phase, two identical objects were placed equidistant from the walls, and mice were allowed to explore for 10 min. After a short interval, one of the objects was replaced with a novel object (differing in shape and colour), and the test was continued for another 10 min. All sessions were video recorded using EthoVision XT 15.0. The arena and objects were cleaned with 20% ethanol between animals. Object exploration time (sec) and frequency were manually scored using Solomon Coder (RRID:SCR_016041).

#### Elevated plus maze test

Anxiety-like behaviour was assessed in a plus-shaped Plexiglas maze elevated 50 cm above the floor. The maze consisted of two open arms (30 × 7 cm) and two closed arms (30 × 7 cm, walls 30 cm high), with a central platform (7 × 7 cm). Mice were placed in the centre of the maze facing a closed arm and allowed to explore for 5 min. Behaviour was recorded and analysed using EthoVision XT 15.0. The apparatus was cleaned with 20% ethanol between trials. Time spent and frequency of visits to each arm were used as outcome measures.

#### Social preference test

Sociability was assessed in a 40 × 40 cm Plexiglas arena containing two cylindrical wire cages placed in opposite corners. During the habituation phase (5 min), the test mouse explored the empty arena. In the test phase, an unfamiliar age- and sex-matched mouse (stranger) was placed in one of the cages, while the other cage remained empty. The test mouse was returned to the arena and allowed to explore for 10 min. Behavior was tracked using EthoVision XT 15.0. Social interaction was defined as time spent sniffing the stranger mouse, manually scored using Solomon Coder (RRID:SCR_016041). The social preference index (SP index) was calculated as:

$$\begin{array}{l}\mathrm{SP}\mathrm{index}=\mathrm{Time}\mathrm{interacting}\mathrm{with}\mathrm{stranger}/\\ (\mathrm{Timewithstranger}+\mathrm{Timewithemptycage}).\end{array}$$

#### Self-grooming test

To assess novelty-induced grooming behaviour, mice were acclimated to the testing room for at least 1 h prior to the test. Each mouse was individually placed into a clear glass observation cylinder (12 cm diameter, 20 cm height) for 10 min, and behaviour was video recorded. The cylinders were cleaned with water between tests. Grooming behaviour was manually scored using Solomon Coder, with the total duration (seconds) and frequency of grooming recorded.

#### Marble burying test

This test was conducted in standard housing cages (36.7 × 14.0 × 20.7 cm) filled with 5 cm of corn cob bedding. Twenty blue glass marbles were arranged in a 4 × 5 grid on the surface, leaving at least 1/5 of the area marble-free. Mice were placed on the marble-free area and allowed to explore for 10 min. Marbles were considered buried if at least 60% of their surface was covered with bedding.

### Multiplex bead array analyses of cytokines

Fetal brain samples and maternal plasma were collected 24 h after drug administration at embryonic day 13.5 (E13.5). Approximately 800 µl of blood was collected from the vena cava of deeply anesthetized pregnant mice (isoflurane until loss of paw withdrawal reflex) using heparinized syringes and a 25 G × 5/8” needle. Blood samples were centrifuged at 12,000 rpm for 10 min at 4 °C, and plasma was collected from the supernatant.

Fetal brains were homogenized in phosphate-buffered saline and centrifuged, and the resulting supernatants were used to assess the levels of inflammatory mediators. The following cytokines were measured using cytometric bead array (CBA) and flow cytometry: IFN-γ (RRID:AB_2869141), IL-1α (RRID:AB_2869318), IL-1β (RRID:AB_2869322), IL-2 (RRID:AB_2869142), IL-4 (RRID:AB_2869143), IL-6 (RRID:AB_2869146), IL-10 (RRID:AB_2869145), IL-17A (RRID:AB_2869329), KC (RRID:AB_2869165), MCP-1 (RRID:AB_2869167) and TNF-α (RRID:AB_2869144). Samples were analyzed with a BD FACSVerse flow cytometer using a commercially available CBA Kit (BD Biosciences, San Jose, CA, US, Cat# 558267). Data were processed using dedicated analysis software (FCAP ARRAY V3 Software (BD Biosciences, San Jose, CA, US, Cat# 652099)), following the manufacturer’s protocol.

Cytokine concentrations were normalized to total protein content, which was determined by photometric measurement using a bicinchoninic acid (BCA) protein assay (Thermo Scientific Pierce, Rockford, IL, US, Cat# 23227). Absorbance was read at 560 nm using a microplate spectrophotometer (Thermo Scientific^TM^, Waltham, Massachusetts, U.S). Plasma cytokine levels are expressed in pg/ml, while fetal brain cytokine levels are expressed in pg/mg protein.

### Enzyme-linked immunosorbent assays

The concentration of soluble P2X7R in maternal plasma was measured using an ELISA kit (MyBiosource, San Diego, CA, USA, Cat# MBS2022553; Aviva Systems Biology, San Diego, CA, USA, Cat# OKEH05605) following the manufacturer’s instruction. Samples were measured in duplicate, and absorbance was read at 450 nm using a microplate spectrophotometer, and sP2X7R concentrations were expressed as pg ml^-1^. The CRP levels were measured in maternal plasma and embryonic brain homogenates using a commercially available Mouse CRP DuoSet Elisa Kit (R&D Systems, Minneapolis, MN, US, Cat# DY1829) according to the manufacturer’s instructions. Absorbance was read at 450 nm, and CRP concentrations were expressed as ng ml^−1^ and pg mg^−1^. The BDNF levels were measured in embryonic brain and adult offspring prefrontal cortex (PFC) and hippocampus (HP) homogenates using a commercially available Human/Mouse BDNF DuoSet ELISA Kit (R&D Systems, Minneapolis, MN, US, Cat# DY248) according to the manufacturer’s instructions. The assay detects total BDNF protein and does not distinguish between proBDNF and mature BDNF isoforms. These samples were not measured in duplicate. Absorbance was read at 450 nm, and BDNF concentrations were expressed as pg mg^−1^.

### TBARS measurement

Lipid peroxidation levels were assessed using a thiobarbituric acid reactive substances (TBARS) assay kit. The protocol was performed according to the manufacturer’s instructions (Sigma-Aldrich, St. Louis, MO, US, Cat# MAK085). Results were expressed as nmol malondialdehyde (MDA) mg^−1^.

### Immunohistochemistry

Fetal heads were collected 24 h after the intraperitoneal injection of dams with either saline, poly(I:C) (10 mg/kg), or the vaccines on E12.5, and samples were immersion fixed in 4% PFA for 24 h at 4 °C. 40 μm coronal sections were cut and blocked in 0.03% Triton-X, 5% normal goat serum and 1% BSA for 1 h at room temperature. Primary Iba1 antibody (1:500, Cat# 019-19741, Wako) was applied overnight at 4 °C; then slices were rinsed and washed three times in PB, incubated with fluorescent secondary antibodies (1:500 Alexa-488 anti-rabbit, Invitrogen) containing 1:10,000 Hoechst 33342 (Cat# 5117, Tocris Bioscience) for 2 h at RT, and rinsed and washed in PB three times again. Slides were covered with Fluoroshield mounting medium (Cat# F6182, Sigma-Aldrich), and the fetal brain was imaged with a confocal Nikon C2 microscope at 10× magnification. Iba1 positive cells were quantified in the dorsal telencephalon and in the ganglionic eminence with ImageJ software.

### Quantification and statistical analysis

Sample size was calculated using G*Power software. Data acquisition and quantification were performed by investigators blinded to the experimental groups. For in vitro experiments, data points were derived from one or two parallel measurements. Statistical analyses were conducted only when each group included at least five independent samples. No data points were excluded as outliers; all values were retained for both analysis and graphical presentation to preserve sample independence and avoid bias introduced by post-hoc data removal. Data are expressed as mean ± SEM.

Normality of data distribution was assessed by the Shapiro-Wilk test. For normally distributed data, one-way ANOVA followed by Tukey’s post hoc test was applied; for non-normally distributed data, Kruskal-Wallis tests with multiple comparisons were used. Post hoc analyses were performed only if ANOVA results showed significance (p < 0.05). Statistical analyses were carried out using STATISTICA version 14.0.1 software (TIBCO Software Inc., Palo Alto, CA, US).

Behavioural data were analysed using Generalized Linear Models (GLMs) with appropriate distributions depending on data type. Count data were analysed using a Poisson distribution with log link, continuous, percentage and index-type data with a Gamma distribution and log link. Sex, Treatment, and their interaction were included as fixed factors. Wald χ² statistics are reported for main and interaction effects, with Tukey-adjusted post hoc tests performed on estimated marginal means. Detailed results are provided in S1 Table. P-values of less than 0.05 were considered statistically significant throughout the study.

Power analysis was performed with G*Power 3.1.9.2 (ANOVA: a priori: compute required sample size; Power: 0.80; α error probability: 0.05; Effect size: 0.3787972; total sample size: 84; sample size: approx. 21 animals/group). Based on previous experience, fecundity rates were approx. 40% after poly(I:C) treatment and 80% after saline treatment, with an average litter size of 7–8 pups per dam; therefore, 15–20 breeding pairs were established for the experiments.

## Results

In a MIA model, we assessed offspring behaviour following maternal BNT162b2 mRNA COVID-19 vaccination. Figure 1 presents a schematic overview of the experimental design, treatment groups, and timeline.

### Behavioural outcomes in offspring following prenatal exposure

Behavioural testing was performed at 4 and 8 weeks of age. Data were analysed using a two-factor Generalized Linear Model (GLZ) including sex, treatment, and their interaction as fixed factors. Detailed statistics and post hoc comparisons are provided in Supplementary Table 1; pooled data are shown in Supplementary Figures S1–S5.

### Open field (OF) test

**Fig. 2:**
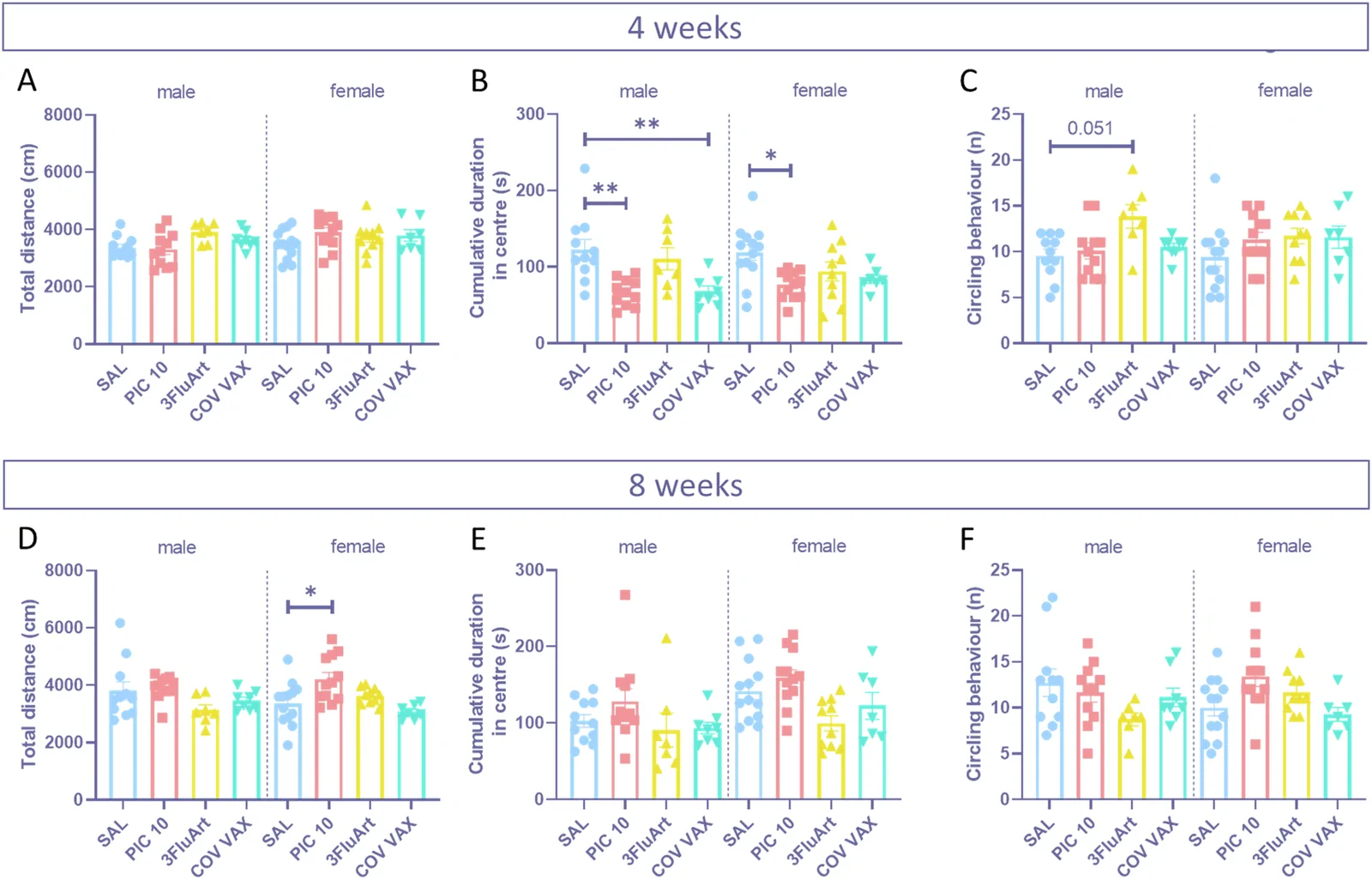
Sex-specific behaviour in open field test at 4 and 8 weeks of age. Panels show data from male and female offspring of four groups: MIA induced by poly(I:C) (PIC 10), saline control (SAL), influenza vaccine (3FluArt), and COVID-19 vaccine (COV VAX). Open field (**A**-**C, D**-**F**): distance travelled (**A**, **D**), time spent in the centre (**B**, **E**), and number of wall circles (**C,** **F**). Values are presented as means ± SEM of n = 15–24 mice per group. Count data (head dips, grooming frequency, and rotation counts) were analysed using Generalized Linear Models (GLZs) with a Poisson distribution and log link, including Treatment, Sex, and their interaction as fixed factors. Continuous and percentage data (e.g., exploration time, intruder sniffing, and other behavioural parameters) were analysed using GLZs with a Gamma distribution and log link, which are appropriate for positive, right-skewed variables. Wald χ² statistics are reported for main and interaction effects, with Tukey-adjusted post hoc tests. Significance was set at p < 0.05. 3FluArt, influenza vaccine, COV VAX, BNT162b2 vaccine, PIC 10, poly(I:C), SAL, saline.

At 4 weeks of age, no significant sex × treatment interaction was detected for total distance travelled or time spent in the centre zone (Fig. 2A). A significant main effect of treatment was observed for centre duration (Wald χ²(3) = 9.12, p < 0.05). Post hoc comparisons revealed reduced centre time in male COV VAX and PIC 10 offspring compared to saline controls (p < 0.01), and in female PIC 10 offspring (p < 0.05) (Fig. 2B).

At 8 weeks, treatment significantly affected total distance travelled (Wald χ²(3) = 9.80, p = 0.0205), driven by increased locomotion in female PIC 10 offspring compared to saline (p < 0.05) (Fig. 2D). Circling behaviour showed a significant main effect of treatment at 8 weeks (Wald χ²(3) = 10.07, p = 0.0180), without significant pairwise differences after correction (Fig. 2F).

### Novel object recognition (NOR) test

**Fig. 3:**
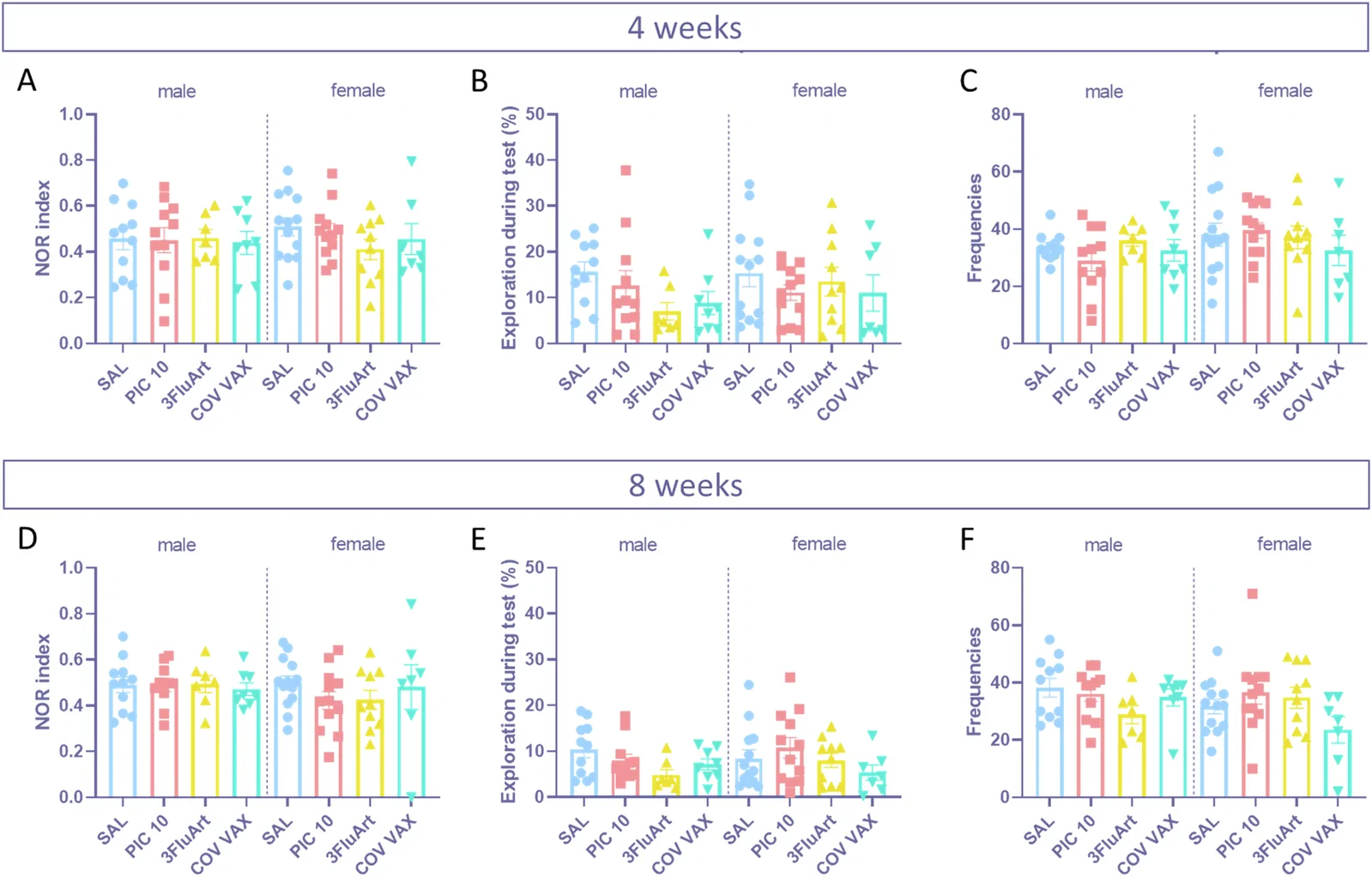
Sex-specific behaviour in novel object recognition test at 4 and 8 weeks of age. Panels show male and female offspring from four groups: MIA induced by poly(I:C) (PIC 10), saline control (SAL), influenza vaccine (3FluArt), and COVID-19 vaccine (COV VAX). Cognitive performance (**A**-**C**, **D**–**F**): NOR index (**A**, **D**), exploration time of novel vs familiar objects (**B**, **E**), number of interactions with objects (**C**, **F**). Values are presented as means ± SEM of n = 15-24 mice per group. Count data (head dips, grooming frequency, and rotation counts) were analysed using Generalized Linear Models (GLZs) with a Poisson distribution and log link, including Treatment, Sex, and their interaction as fixed factors. Continuous and percentage data (e.g., exploration time, intruder sniffing, and other behavioural parameters) were analysed using GLZs with a Gamma distribution and log link, which are appropriate for positive, right-skewed variables. Wald χ² statistics are reported for main and interaction effects, with Tukey-adjusted post hoc tests. Significance was set at p < 0.05. 3FluArt, influenza vaccine, COV VAX, BNT162b2 vaccine, PIC 10, poly(I:C), SAL, saline.

No significant sex × treatment interactions were observed for recognition index or exploration time at either age (Fig. 3A, B, D, E).

The number of object interactions showed a significant main effect of treatment at 4 weeks (Wald χ²(3) = 10.10, p = 0.0177) and 8 weeks (Wald χ²(3) = 23.39, p = 0.000034); however, post hoc pairwise comparisons did not identify specific group differences (Fig. 3C, F).

### Elevated plus maze (EPM) test

**Fig. 4:**
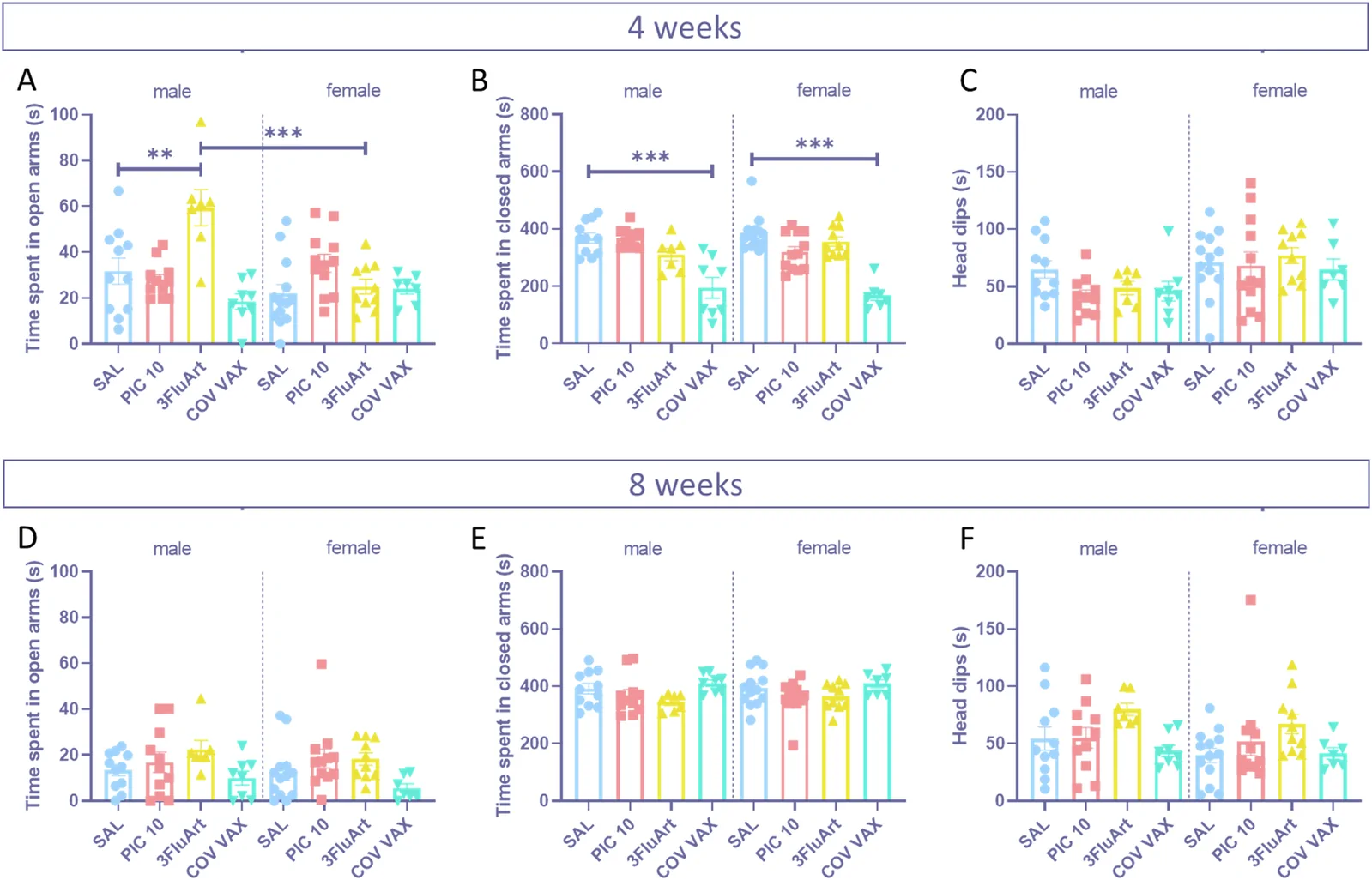
Sex-specific behaviour in elevated plus maze test at 4 and 8 weeks of age. Panels show male and female offspring from four groups: MIA induced by poly(I:C) (PIC 10), saline control (SAL), influenza vaccine (3FluArt), and COVID-19 vaccine (COV VAX). Anxiety-related behaviour (**A**-**C**, **D**–**F**): time in open/bright arms (**A**, **D**), time in closed/dim arms (**B**, **E**), and number of head dips (**C**, **F**). Values are presented as means ± SEM of n = 15-24 mice per group. Count data (head dips, grooming frequency, and rotation counts) were analysed using Generalized Linear Models (GLZs) with a Poisson distribution and log link, including Treatment, Sex, and their interaction as fixed factors. Continuous and percentage data (e.g., exploration time, intruder sniffing, and other behavioural parameters) were analysed using GLZs with a Gamma distribution and log link, which are appropriate for positive, right-skewed variables. Wald χ² statistics are reported for main and interaction effects, with Tukey-adjusted post hoc tests. Significance was set at p < 0.05. 3FluArt, influenza vaccine, COV VAX, BNT162b2 vaccine, PIC 10, poly(I:C), SAL, saline.

At 4 weeks, a significant sex × treatment interaction was detected for time spent in the open arms (Wald χ²(3) = 10.36, p = 0.0157) (Fig. 4A). Post hoc analysis revealed that male 3FluArt offspring spent more time in the open arms compared to male saline controls (p < 0.01), whereas no such effect was observed in females. Additionally, female 3FluArt offspring spent significantly less time in the open arms than their male counterparts (p = 0.00019) (Fig. 4A).

Time spent in closed arms showed a significant main effect of treatment at 4 weeks, with reduced duration in both male and female COV VAX offspring (male p < 0.001; female p < 0.001), without a significant interaction (Fig. 4B).

At 8 weeks, no significant sex × treatment interaction was observed for open-arm or closed-arm time (Fig. 4D, E).

Head-dipping behaviour did not show consistent sex × treatment effects at either age after post hoc correction (4-week-old: Wald χ²(3) = 34.64, p < 0.000001; post hoc ns; 8-week-old: Wald χ²(3) = 11.39, p = 0.0098; post hoc ns) (Fig. 4C, F).

### Social preference (SP) test

**Fig. 5:**
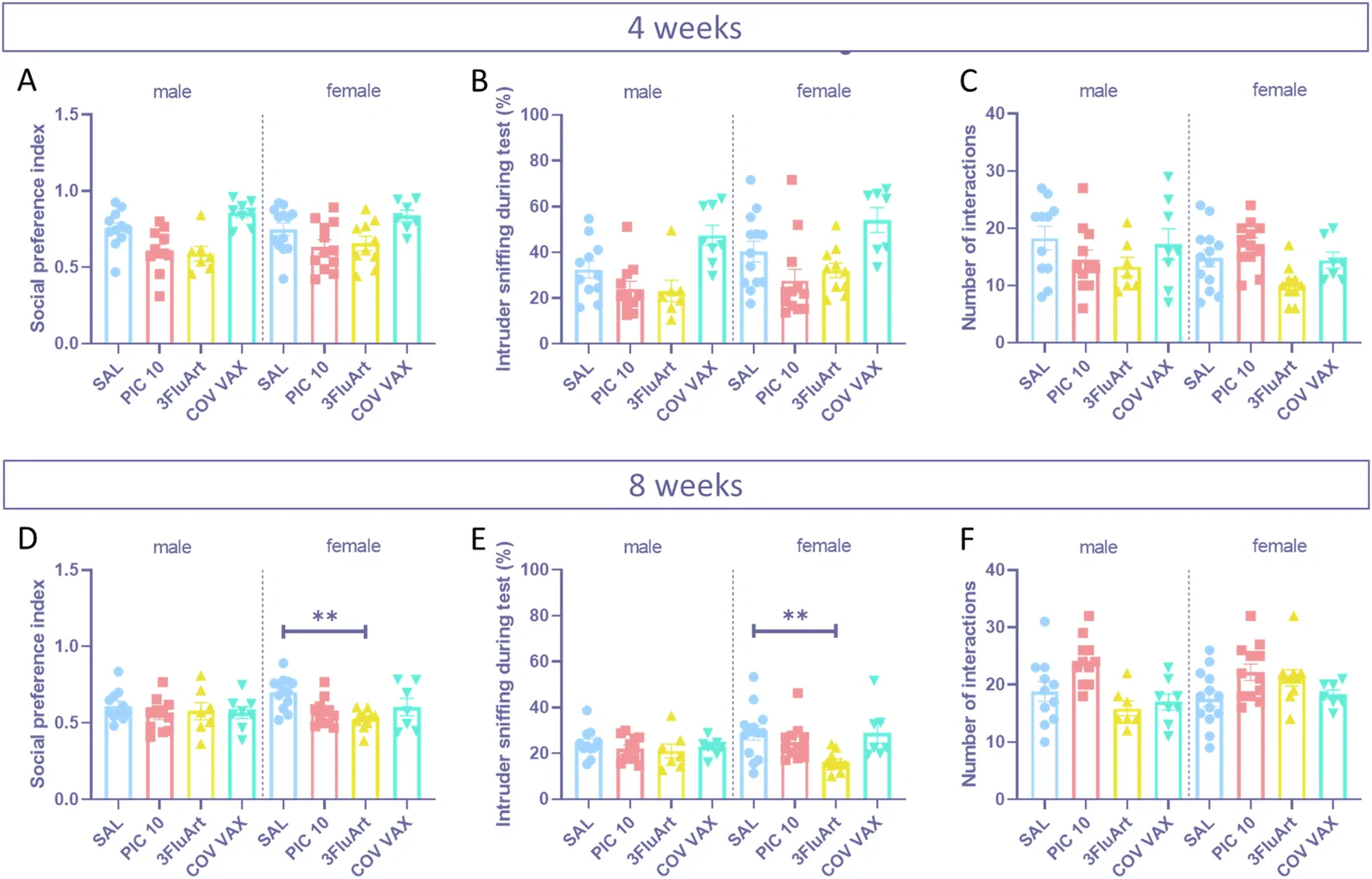
Sex-specific behaviour in social preference test at 4 and 8 weeks of age. Panels show male and female offspring from four groups: MIA induced by poly(I:C) (PIC 10), saline control (SAL), influenza vaccine (3FluArt), and COVID-19 vaccine (COV VAX). Social preference index (**A**, **D**), intruder sniffing (%) (**B**, **E**), and number of interactions with stranger mouse (**C**, **F**). Values are presented as means ± SEM of n = 15-24 mice per group. Count data (head dips, grooming frequency, and rotation counts) were analysed using Generalized Linear Models (GLZs) with a Poisson distribution and log link, including Treatment, Sex, and their interaction as fixed factors. Continuous and percentage data (e.g., exploration time, intruder sniffing, and other behavioural parameters) were analysed using GLZs with a Gamma distribution and log link, which are appropriate for positive, right-skewed variables. Wald χ² statistics are reported for main and interaction effects, with Tukey-adjusted post hoc tests. Significance was set at p < 0.05. 3FluArt, influenza vaccine, COV VAX, BNT162b2 vaccine, PIC 10, poly(I:C), SAL, saline.

At 4 weeks, a significant main effect of treatment was detected for the number of social interactions (Wald χ²(3) = 12.46, p = 0.0059), without a significant interaction. Post hoc comparisons did not reveal specific group differences (Fig. 5C).

At 8 weeks, treatment approached significance for interaction number (Wald χ²(3) = 7.81, p = 0.050) (Fig. 5F), and female 3FluArt offspring showed reduced social preference index and stranger sniffing duration compared to saline females (p < 0.01) (Fig. 5D, E).

### Self-grooming and marble burying (MB) test

**Fig. 6:**
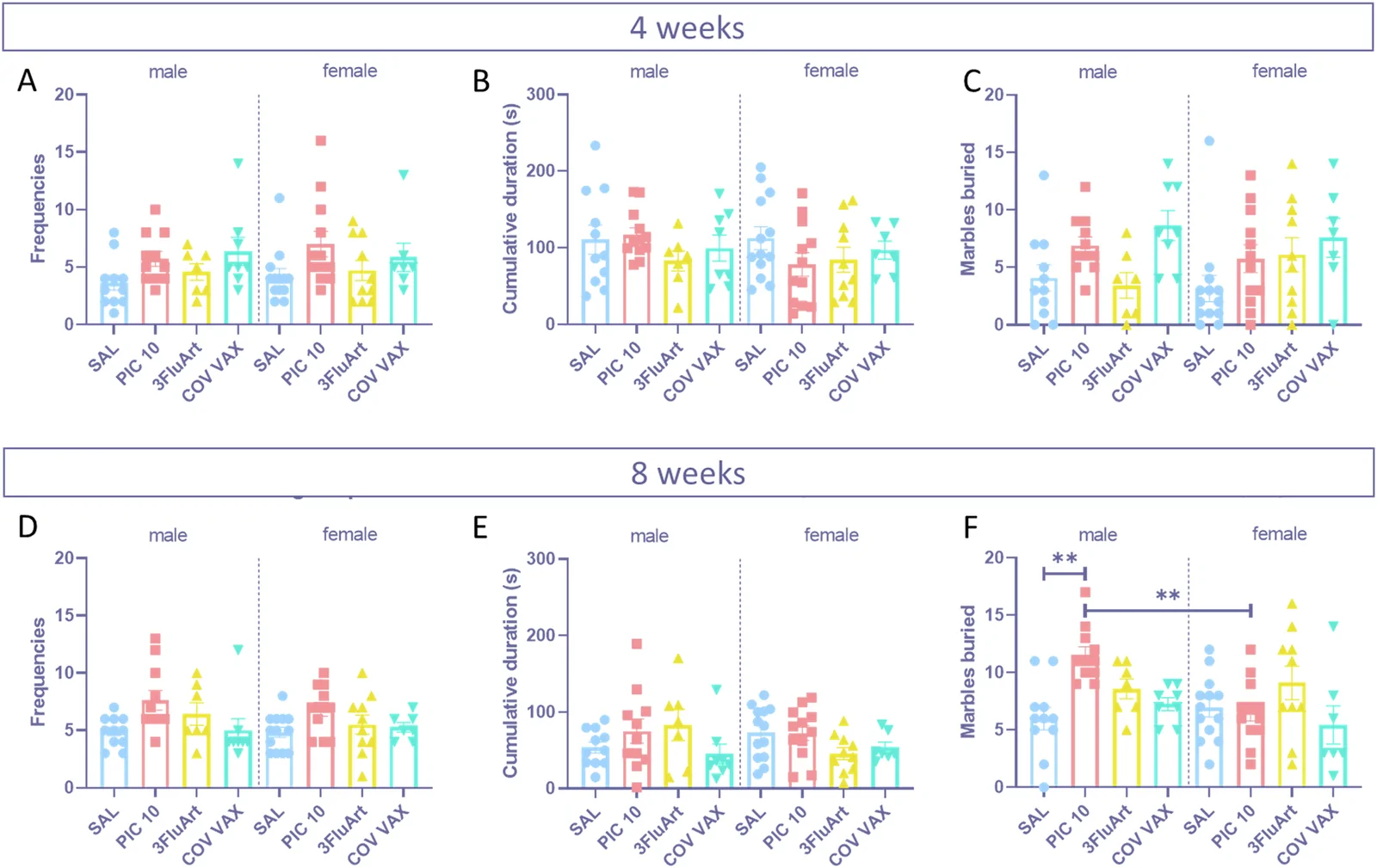
Sex-specific behaviour in self-grooming and marble burying tests at 4 and 8 weeks of age. Panels show male and female offspring from four groups: MIA induced by poly(I:C) (PIC 10), saline control (SAL), influenza vaccine (3FluArt), and COVID-19 vaccine (COV VAX). Self-grooming and marble burying (**A**, **B**, **D**, **E**, and **C**, **F**): grooming frequency (**A**, **D**), cumulative grooming duration (**B**, **E**), and number of buried marbles (**C**, **F**). Values are presented as means ± SEM of n = 15-24 mice per group. Count data (head dips, grooming frequency, and rotation counts) were analysed using Generalized Linear Models (GLZs) with a Poisson distribution and log link, including Treatment, Sex, and their interaction as fixed factors. Continuous and percentage data (e.g., exploration time, intruder sniffing, and other behavioural parameters) were analysed using GLZs with a Gamma distribution and log link, which are appropriate for positive, right-skewed variables. Wald χ² statistics are reported for main and interaction effects, with Tukey-adjusted post hoc tests. Significance was set at p < 0.05. 3FluArt, influenza vaccine, COV VAX, BNT162b2 vaccine, PIC 10, poly(I:C), SAL, saline.

Grooming duration and frequency did not show significant sex × treatment interactions at either age (Fig. 6A, B, D, E).

In the marble burying test, a significant sex × treatment interaction was observed at 4 weeks (Wald χ²(3) = 8.53, p = 0.0362), although post hoc comparisons were not significant (Fig. 6C).

At 8 weeks, the interaction remained significant (Wald χ²(3) = 13.43, p = 0.0037), driven by increased marble burying in male PIC 10 offspring compared to saline (p < 0.01) and compared to female PIC 10 offspring (p = 0.0077) (Fig. 6F).

Overall, prenatal vaccination was associated with mild, age- and sex-dependent behavioural alterations, whereas poly(I:C) exposure produced more pronounced effects. The pooled data analysis (Fig S1-S5) confirms this observation showing that in contrast to poly(I:C) treatment, which elicited the well-known behavioural alterations mimicking autism-like behaviours, prenatal vaccination only slightly replicated these effects.

### Prenatal experiments

**Fig. 7:**
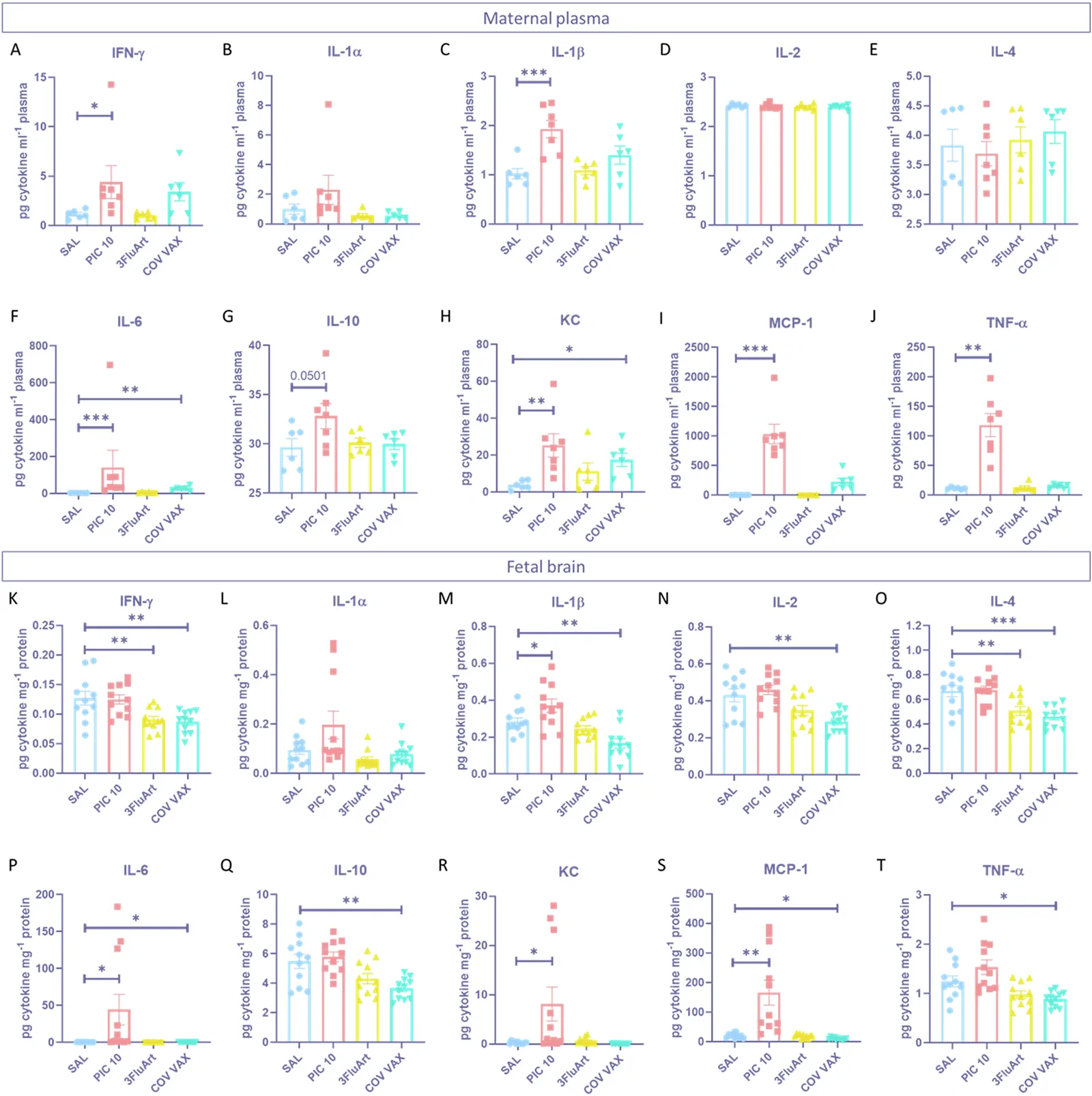
Cytokine and chemokine levels in the maternal plasma and fetal brain 24 h after injections on the day 13.5 of gestation. The expression of pro- and anti-inflammatory cytokine and chemokine levels was measured by CBA FACS analysis in pregnant mice. Values are presented as means ± SEM of n = 6-8 mice per group. Depending on the normal distribution of the data set, one-way ANOVA with Dunnett’s multiple comparison *post hoc* (**C**, **D**, **G**, **K**, **M**, **N**, **O**, **Q**, **T**) or Kruskal-Wallis with multiple comparisons (**A**, **B**, **E**, **F**, **H**, **I**, **J**, **L**, **P**, **R**, **S**): ^*^p < 0.05. 3FluArt, influenza vaccine, COV VAX, BNT162b2 vaccine, IFN, interferon; IL, interleukin, KC, keratinocyte-derived chemokine, PIC 10, poly(I:C), SAL, saline, MCP-1, monocyte chemoattractant protein-1, TNF, tumour necrosis factor.

Levels of interferon (IFN)-γ, interleukin (IL)-1α, IL-1β, IL-2, IL-6, IL-10, keratinocyte-derived chemokine (KC), monocyte chemoattractant protein (MCP)-1 and tumour necrosis factor (TNF)-α were measured by CBA FACS analysis in maternal plasma and embryonic brain 24 h after injection on gestational day 13.5 (Fig. 7).

A robust immune response was observed in maternal plasma following both poly(I:C) and COVID-19 vaccine administration. Poly(I:C) significantly increased the levels of IFN-γ, IL-1β, IL-6, IL-10, KC, MCP-1 and TNF-α (Fig. 7A, C, F–J). In contrast, COVID-19 vaccine caused a moderate increase in IL-6 and KC levels only (Fig. 7F, KC). Thus, poly(I:C) induced a stronger maternal cytokine response than the mRNA vaccine.

In the fetal brain, maternal poly(I:C) exposure led to increased levels of IL-1β, IL-6, KC and MCP-1 in their brain (Fig. 7M, P, R, S). By contrast, cytokine levels in the brains of ‘COV VAX’ foetuses were generally lower than those of the ‘SAL’ group, including IFN-γ, IL-1β, IL-2, IL-6, IL-10, MCP-1 and TNF-α (Fig. 7K, M, N, O, P, Q, S, T).

**Fig. 8:**
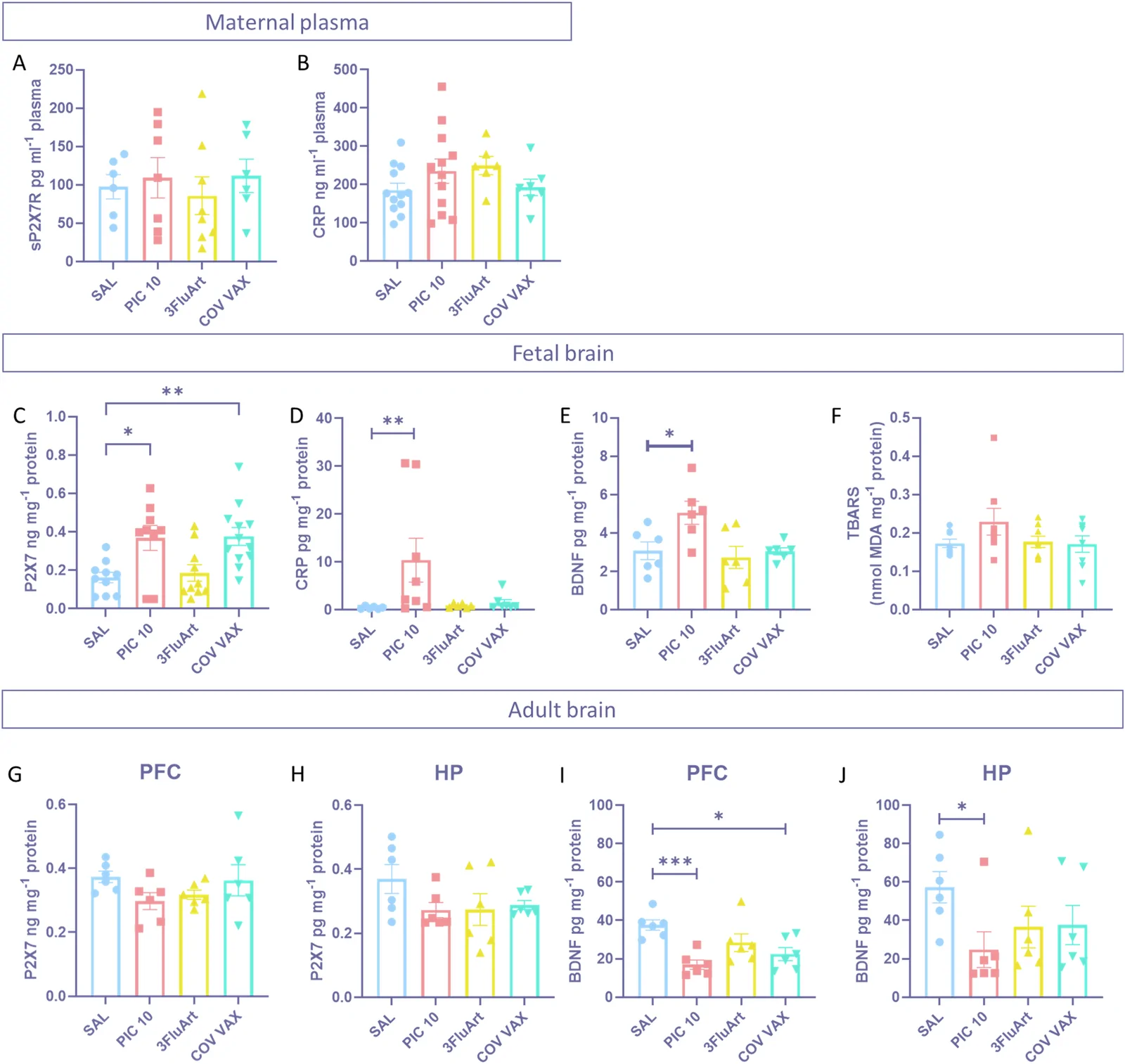
Maternal, fetal and adult offspring markers following prenatal treatments. Maternal plasma sP2X7R and CRP levels (**A**, **B**) and fetal brain P2X7R, CRP, BDNF and TBARS levels in (**C**, **D**, **E**, **F**) 24 h after injections on the day 13.5 of gestation, and adult offspring P2X7R, BDNF levels in prefrontal cortex (PFC) and hippocampus (HP) (**G**, **H**, **I**, **J**). The expression of CRP, total BDNF and P2X7R levels was measured by ELISA, TBARS were measured using lipid peroxidation (MDA) assay kit. Values are presented as means ± SEM of n = 6–12 mice per group. Depending on the normal distribution of the data set, one-way ANOVA with Dunnett’s multiple comparison *post hoc* (**A,** **B,** **C,** **E,** **G,** **H,** **I**) or Kruskal-Wallis with multiple comparisons (**D**, **F**, **J**): ^*^p < 0.05. 3FluArt, influenza vaccine, BDNF, brain-derived neurotrophic factor, COV VAX, COVID-19 BNT162b2 vaccine, CRP, C-reactive protein, PIC 10, poly(I:C), SAL, saline, sP2X7R, soluble P2X7 receptor, TBARS, thiobarbituric acid reactive substances.

Soluble P2X7 receptor (sP2X7R) and C-reactive protein (CRP) levels were quantified in maternal plasma 24 h after treatment on gestational day 13.5 (Fig. 8A, B). Maternal sP2X7R concentrations did not differ between groups, indicating that neither the vaccines nor poly(I:C) altered circulating soluble receptor levels (Fig. 8A). Maternal CRP levels showed a mild, non-significant elevation in both the ‘PIC 10’ and ‘3FluArt’ groups relative to the ‘SAL’ controls, while the ‘COV VAX’ group remained comparable to baseline (Fig. 8B).

Fetal brain inflammatory and neurotrophic markers were also assessed at the same time point (Fig. 8C–F). Fetal P2X7 receptor levels were significantly increased in the ‘PIC 10’ (p < 0.05) and ‘COV VAX’ (p < 0.01) groups compared with ‘SAL’ (Fig. 8C). Fetal CRP concentrations were significantly increased in the ‘PIC 10’ group (p < 0.01) compared with ‘SAL’, revealing a pronounced inflammatory response in the offspring despite only moderate CRP changes in maternal plasma (Fig. 8D). In contrast, fetal CRP levels in the ‘COV VAX’ and ‘3FluArt’ groups did not differ from controls (Fig. 8D). Fetal BDNF levels were markedly elevated following ‘PIC 10’ administration, while no changes were detected after ‘SAL’, ‘COV VAX’, or ‘3FluArt’ treatment (Fig. 8E). Oxidative stress, indexed by TBARS levels in the fetal brain, showed a slight, non-significant increase in the ‘PIC 10’ group, with all other groups remaining comparable to controls (Fig. 8F).

To examine potential long-term neurobiological effects, P2X7 receptor levels were measured in adult offspring in the prefrontal cortex (PFC) and hippocampus (HP) (Fig. 8G, H). When males and females were pooled, no significant differences were detected in any experimental group compared with ‘SAL’ in either brain region. Sex-specific analyses of P2X7 receptor levels in the PFC and HP are presented separately in the supplementary material (S7 Fig).

BDNF protein levels were also assessed in adult offspring. In the PFC, BDNF levels were significantly reduced in both the ‘PIC 10’ (p < 0.001) and ‘COV VAX’ (p < 0.05) groups relative to ‘SAL’, whereas the ‘3FluArt’ group showed no alteration (Fig. 8I). In the hippocampus, BDNF concentrations were significantly decreased only in the ‘PIC 10’ group (p < 0.05), while the ‘COV VAX’ and ‘3FluArt’ groups did not differ from controls (Fig. 8J). Additionally, sex-specific BDNF levels in the PFC and HP are presented separately in the supplementary material (S8 Fig).

**Fig. 9:**
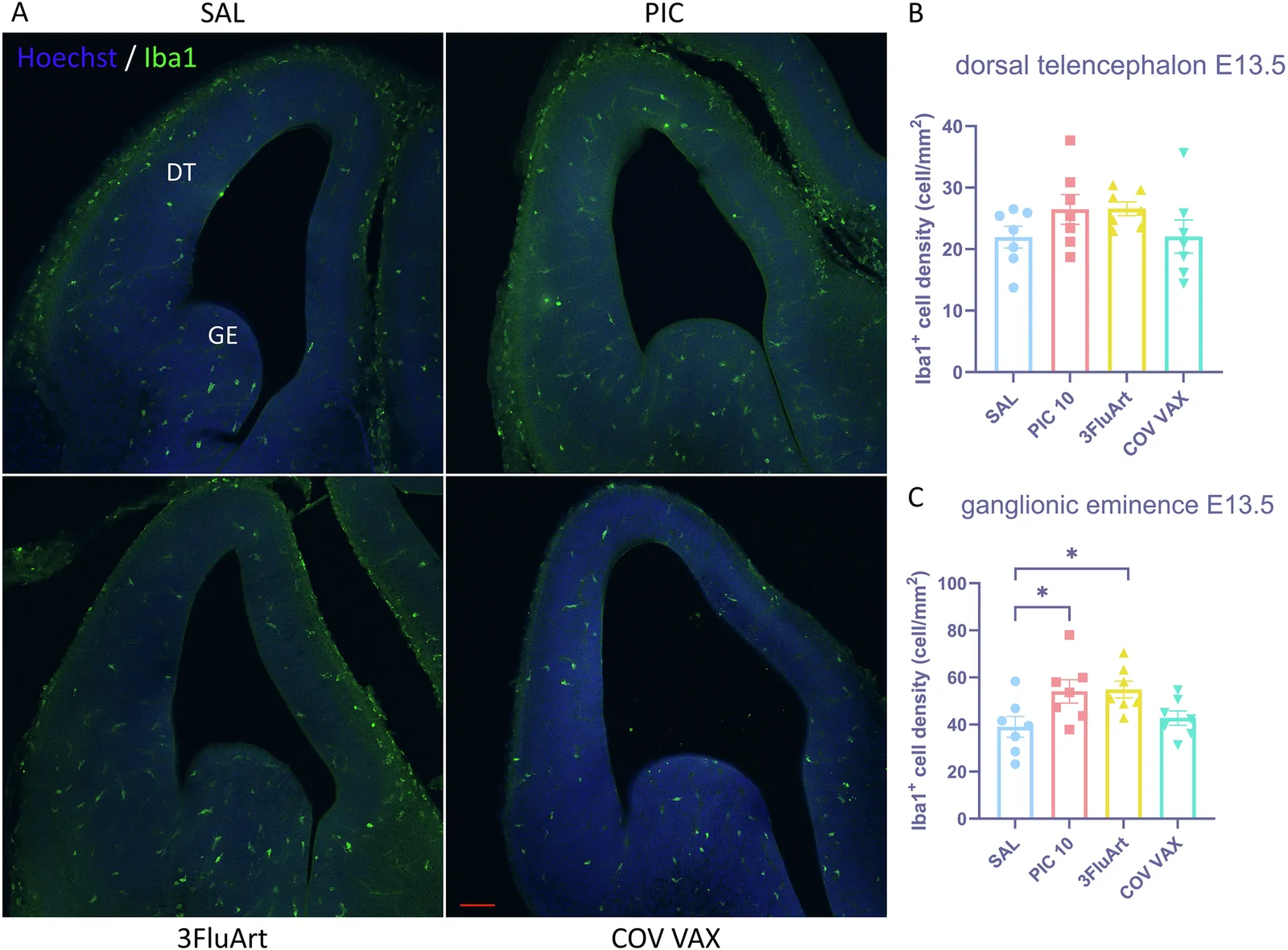
Iba1 immunostaining in the embryonic brain at E13.5. (**A**) Representative images of Iba1 immunostaining (green) in the dorsal telencephalon and ganglionic eminence from SAL, PIC 10, 3FluArt, and COV VAX groups. All images were acquired using identical microscope settings (laser intensity, gain, and exposure parameters) across all experimental groups. (**B**) Quantification of Iba1⁺ cell density (cells/mm²) in the dorsal telencephalon. (**C**) Quantification of Iba1⁺ cell density (cells/mm²) in the ganglionic eminence. Values are presented as means ± SEM of n = 7 mice per group. Depending on the normal distribution of the data set, one-way ANOVA with Dunnett’s multiple comparison *post hoc* (B, C): ^*^p < 0.05. 3FluArt, influenza vaccine, COV VAX, COVID-19 BNT162b2 vaccine, DT, dorsal telencephalon, GE, ganglionic eminence, Hoechst, nuclei labelling fluorescent DNA stain, Iba1^+^, ionized calcium-binding adapter molecule 1 microglial marker, PIC 10, poly(I:C), SAL, saline, sP2X7R, soluble P2X7 receptor, TBARS, thiobarbituric acid reactive substances.

Iba1 immunostaining was performed to assess microglial cell density in the embryonic brain at E13.5 (Fig. 9). In the dorsal telencephalon, Iba1⁺ cell density (cells/mm²) was increased in the PIC 10 and 3FluArt groups compared to the saline (SAL) group; however, these differences did not reach statistical significance. No change was observed in the COV VAX group relative to SAL (Fig. 9B). In the ganglionic eminence, Iba1⁺ cell density was significantly increased in both the PIC 10 and 3FluArt groups compared to SAL. In contrast, the COV VAX group did not differ from the SAL group (Fig. 9C).

Taken together, maternal poly(I:C) exposure elicited a robust systemic immune response accompanied by marked inflammatory and neurotrophic alterations in the fetal brain, including increased cytokines, CRP, P2X7 receptor levels and region-specific microglial changes, with persistent reductions in adult BDNF expression.

In contrast, prenatal mRNA COVID-19 vaccination induced a moderate maternal cytokine response without overt fetal neuroinflammation or microglial activation but was associated with elevated fetal P2X7 receptor levels and a selective reduction of BDNF in the adult prefrontal cortex. Influenza vaccination produced only limited and largely transient effects.

## Discussion

In this study we investigated the long-term neurobiological and behavioural effects of prenatal exposure to maternal BNT162b2 COVID-19 mRNA vaccination in mice. While the immunogenicity and short-term safety of BNT162b2 are well characterised, data on immunisation during pregnancy remain limited. Pregnant women face higher risks of severe illness, respiratory complications, and preterm birth due to COVID-19.

Vaccination remains one of the most effective tools for preventing viral infections and related complications. However, excessive maternal immune stimulation during pregnancy has been linked to neurodevelopmental disorders, including ASD [29]. Animal studies have shown that MIA correlates with brain changes and behavioural deficits in offspring. Previous work has described embryonic and postnatal immunogenicity following maternal COVID-19 vaccination [21, 24].

Poly(I:C) administration is a well-known rodent model of mimicking maternal viral infection inducing ASD-like characteristics in offspring [6, 11]. Our goal was to investigate the long-term effects of prenatal immunisation with BNT162b2 COVID-19 mRNA vaccine and 3FluArt influenza vaccine. Unlike poly(I:C), which mimics viral infection and induces robust MIA-like outcomes, maternal vaccination did not produce a consistent or persistent autism-relevant behavioural profile. This contrast allowed us to distinguish coordinated inflammatory programming from selective and transient immune modulation. This distinction gains further relevance in light of recent epidemiological data suggesting that maternal SARS-CoV-2 infection during the first or second trimester may elevate ASD risk in female offspring [30]. This underscores the need to differentiate infection-associated immune activation from vaccine-related immune modulation.

Consistent with established maternal immune activation (MIA) paradigms, poly(I:C) exposure produced robust and reproducible behavioural alterations across domains. Offspring displayed increased locomotor activity, anxiety-related behaviour (S1 Fig), and alterations in repetitive and social measures (S4 and S5 Figs), in line with prior reports demonstrating that strong prenatal innate immune activation can induce long-lasting behavioural phenotypes relevant to neurodevelopmental disorders. These effects were observed across ages. Sex-dependent modulation was consistent with literature describing sexually dimorphic vulnerability to prenatal inflammatory insults [10, 11, 31–33]. Maternal inflammation has been suggested to exert sex-specific effects on the risk of psychosis or depression, and brain function, in adult offspring [34–37]. In our study, male offspring exposed to poly(I:C) showed increased repetitive behaviour at 8 weeks, while females did not (Fig. 6F). In addition, female—but not male—poly(I:C) offspring exhibited increased total distance travelled in the open field at 8 weeks, indicating a sex-dependent alteration in locomotor activity (Fig. 2D). Nonetheless, both sexes displayed autistic-like traits in sociability tasks.

In contrast, prenatal exposure to the BNT162b2 COVID-19 vaccine resulted in comparatively mild and largely transient behavioural changes. At 4 weeks of age, reduced centre exploration in the open field suggested increased anxiety-like behaviour, particularly in males, but this effect did not persist into young adulthood (Fig. 2B, E). In the elevated plus maze, offspring spent less time in the closed arms compared to saline controls. However, this was not accompanied by increased open-arm exploration (Fig. 4A, B). This pattern does not indicate a classical anxiolytic profile but rather suggests subtle alterations in risk-assessment or exploratory behaviour. Social interaction and repetitive measures did not show a consistent or sustained impairment across ages. In addition, several statistically significant main effects were not supported by post hoc comparisons or were not consistently replicated across behavioural assays, further reinforcing the absence of a robust behavioural phenotype. Importantly, these behavioural findings occurred in the absence of maternal cytokine elevation, fetal CRP induction, fetal BDNF alteration, or microglial density changes, arguing against coordinated MIA-like neurodevelopmental programming following maternal COVID-19 vaccination.

Interestingly, maternal influenza vaccination (3FluArt) was associated with subtle yet reproducible behavioural modulation. At 4 weeks, male offspring exhibited increased open-arm exploration in the elevated plus maze, indicating altered anxiety-related behaviour (Fig. 4A). Social measures showed modest reductions at later time points, particularly in adult females, as reflected by decreased social preference index and reduced intruder-sniffing duration (Fig. 5A, B). These age- and sex-dependent effects did not constitute a consistent autism-relevant phenotype. Moreover, they occurred without detectable maternal cytokine elevation, fetal CRP increase, or changes in fetal BDNF or soluble P2X7 receptor levels.

A possible interpretation is that aluminium-adjuvanted, multivalent influenza vaccines can elicit low-grade or transient innate immune signalling [38, 39]. This response is insufficient to induce a classical MIA-like neurodevelopmental trajectory, but may still modulate early-life emotional or social behaviour in a mild, age- and sex-dependent manner [40–42]. Such mild immune modulation may influence early neurodevelopment without triggering robust neuroinflammatory cascades. The absence of fetal cytokine induction supports a mechanism distinct from classical MIA.

Further work will be required to clarify these effects. This should include placental analyses and cell-specific neuroimmune markers to determine whether they reflect adjuvant-driven innate immune modulation [38, 39], altered maternal sickness behaviour [43], or stochastic developmental variation.

Sex-dependent patterns observed in both vaccination groups further support the concept that prenatal immune perturbations interact with sexually dimorphic neurodevelopmental programs. While poly(I:C) induced broad behavioural alterations across sexes, vaccination-related effects were modest and partially restricted to specific sex–age combinations. This aligns with accumulating evidence that male and female brains may differ in immune sensitivity and developmental timing, leading to differential behavioural outcomes following prenatal immune challenges.

Strong maternal immune activation induced persistent multi-domain behavioural alterations, whereas maternal vaccination resulted only in mild, transient modulation.

Investigating neuroinflammation and its impact on brain development and synaptic plasticity is essential for understanding the long-term consequences of COVID-19 infection and vaccination. We chose to measure specific cytokines as they are key markers of neuroinflammation and provide insight into the inflammatory responses potentially induced by vaccines. Inflammation can lead to alterations in behaviour and neurodevelopment in the offspring as well. Following MIA, poly(I:C) increased maternal plasma levels of IFN-γ, IL-1β, IL-6, IL-10, KC, MCP-1 and TNF-α. It also elevated IL-1β, IL-6, KC and MCP-1 in fetal brains compared to controls (Fig. 7). In the fetal brain, several cytokines (including IL-1β, IL-2, IL-4, IL-6, IL-10, MCP-1 and TNF-α) were significantly reduced following maternal BNT162b2 vaccination. This finding contrasts with the robust cytokine elevations observed after poly(I:C)-induced maternal immune activation. Beyond their inflammatory role, these cytokines regulate physiological neurodevelopment, including microglial maturation, neuronal differentiation and synaptic refinement. Reduced fetal levels may therefore reflect transient neuroimmune modulation rather than suppression of pathology. However, the functional significance of these reductions remains to be determined. Notably, these alterations were not accompanied by changes in fetal BDNF, oxidative stress markers (TBARS), or soluble P2X7 receptor levels, nor were they associated with persistent behavioural abnormalities in adulthood. Together, these data indicate subtle modulation of the fetal neuroimmune milieu. No evidence of coordinated inflammatory activation was observed. The 3FluArt vaccine triggered no maternal cytokine response, and only IL-4 and IFN-γ levels were slightly altered in fetal brains. Cabău et al [44] described non-specific plasma circulating inflammatory markers after BNT162b2 vaccination in humans, where they also measured no significant changes in the plasma inflammatory proteome after the first few days of administration. Other studies showed normalisation of the inflammatory markers [22, 45], and one reported a prompt short-term increase in serum chemokines and cytokines within 24 h following the vaccination [46].

In addition to cytokine and neurotrophic profiling, we assessed purinergic signalling by quantifying soluble P2X7 receptor (sP2X7R) in maternal plasma and total P2X7 receptor levels in fetal brain and adult offspring regions (Fig. 8). The P2X7 receptor is a key ATP-gated ion channel involved in inflammasome activation, caspase-1 cleavage, and IL-1β release, and has been implicated in maternal immune activation (MIA) models and neurodevelopmental disorders. Maternal circulating sP2X7R levels were unchanged across all groups 24 h after treatment. Because soluble P2X7R reflects receptor shedding and extracellular release rather than membrane-bound functional receptor activity, the absence of changes does not exclude local receptor activation but indicates that systemic inflammasome-associated receptor shedding was not robustly induced under our experimental conditions. Importantly, changes in receptor expression levels do not necessarily reflect functional activation or downstream signalling.

In the fetal brain, P2X7 receptor levels were significantly increased in both the poly(I:C) and the BNT162b2 groups (Fig. 8C). The elevation observed after poly(I:C) is consistent with previous evidence linking MIA to enhanced purinergic danger signalling in the developing brain. P2X7 activation is known to facilitate IL-1β maturation via NLRP3 inflammasome engagement and has been shown to contribute to behavioural abnormalities in MIA paradigms. Genetic deletion or pharmacological inhibition of P2X7 partially prevents autism-relevant behavioural phenotypes in poly(I:C)-exposed offspring, supporting its mechanistic involvement in inflammation-driven neurodevelopmental alterations [10, 11, 47].

Notably, fetal P2X7 levels increased after BNT162b2 exposure despite the absence of broader inflammatory activation (Fig. 8C). This finding is consistent with selective ATP-sensitive signalling modulation rather than coordinated inflammasome programming. This pattern suggests that prenatal vaccination may transiently modulate ATP-sensitive signalling pathways in the fetal brain. However, it does not indicate activation of a full inflammatory cascade. Notably, P2X7 receptor expression can be regulated independently of overt cytokine storms and may respond to subtle shifts in extracellular ATP dynamics or microenvironmental cues during neurodevelopment.

Importantly, these fetal alterations did not persist into adulthood. In pooled male and female offspring, no significant differences in P2X7 receptor levels were detected in the prefrontal cortex or hippocampus (Fig. 8G, H). This absence of long-term purinergic dysregulation contrasts with the persistent neurotrophic and behavioural alterations observed after poly(I:C) exposure and argues against sustained inflammasome-associated reprogramming following maternal vaccination.

Taken together, while poly(I:C) induced a coordinated inflammatory signature including fetal CRP elevation, BDNF increase and P2X7 upregulation, maternal BNT162b2 vaccination was associated only with a selective and transient increase in fetal P2X7 receptor levels without broader neuroinflammatory activation or long-term behavioural sequelae.

In addition to cytokines, we assessed C-reactive protein (CRP) as a broader marker of systemic inflammatory activation. Maternal plasma CRP showed a modest, non-significant elevation after poly(I:C) and 3FluArt exposure (Fig. 8B). Although CRP is a classical acute-phase reactant, evidence indicates that its maternal levels during pregnancy can vary substantially depending on the nature and intensity of the immune challenge [48]. Consistent with previous reports that strong maternal immune activation can induce fetal acute-phase responses, including elevated fetal CRP or related acute-phase markers [49], we observed significantly increased CRP levels in fetal brains following poly(I:C) exposure (Fig. 8D). In contrast, neither COVID-19 nor influenza vaccination increased maternal or fetal CRP levels. This pattern aligns with human studies showing that clinically administered influenza vaccination in pregnancy induces minimal or no CRP elevation compared with infectious stimuli [50, 51]. Together, these findings indicate that maternal vaccination does not elicit a measurable acute-phase response in the maternal–fetal unit, in contrast to experimental viral-mimetic immune activation. Experimental studies in mice show that CRP elevation following inflammatory challenges such as LPS is transient and strongly time-dependent. Levels can return toward baseline within 24 h, depending on dose and experimental context [52].

BDNF levels are low during early embryogenesis and rise progressively toward adulthood, reflecting their key role in synaptic maturation and neurodevelopment [53]. Abnormally elevated BDNF during fetal development has been associated with altered synaptic plasticity and neurodevelopmental abnormalities, including autism spectrum-related phenotypes [54–58]. In our study, poly(I:C) significantly increased fetal brain BDNF levels, whereas neither 3FluArt nor BNT162b2 vaccination altered BDNF expression compared with saline controls (Fig. 8E). This pattern is consistent with other maternal immune activation (MIA) models, in which robust inflammatory signalling—such as LPS exposure—can lead to altered BDNF levels during later postnatal development [5]. One plausible pathway involves maternal IL-6 signalling: maternal IL-6 can engage placental IL-6 pathways and, depending on gestational timing, traverse the placenta to impact the fetal compartment [59]. Neuronal STAT3 activation downstream of IL-6 induces synaptogenic gene programs, including Rgs4, and increases excitatory synapse density [60], while placental IL-6 signalling mediates fetal brain transcriptional changes in MIA models [61]. Other cytokines elevated after poly(I:C), including IL-1β, IFN-γ and TNF-α, also modulate BDNF production in neural and glial cells [62], offering additional direct pathways. Microglial and astrocytic activation in response to maternal immune activation can further alter local neurotrophin expression and synaptic pruning, and recent evidence shows altered proBDNF/BDNF balance in fetal brain after poly(I:C) [63], while placenta-derived neurotrophins or altered placental transport may contribute to changes in fetal BDNF availability. Importantly, these processes are time-dependent and may produce transient increases in fetal BDNF that do not necessarily persist into adulthood; longitudinal and cell-type specific follow-up studies are therefore required to resolve causality and functional consequences.

In contrast, maternal vaccination produced only mild and transient cytokine changes and did not trigger microglial density changes suggestive of altered neuroimmune development rather than direct evidence of microglial activation. This likely explains the absence of fetal BDNF elevation following vaccination. Notably, recent evidence suggests that SARS-CoV-2 infection and immune activation can modulate neurotrophin signaling, including BDNF, potentially contributing to long-term neuropsychiatric outcomes [64]. Moreover, preclinical studies indicate that other types of vaccination or immune stimulation—such as BCG immunization—can alter hippocampal BDNF expression and related neuroplasticity pathways in rodents, supporting the concept that immune challenges during development can transiently influence neurotrophic signalling [65, 66].

To determine whether early-life immune exposures produced delayed neurotrophic alterations, we assessed BDNF levels in the adult offspring brain regions (Fig. 8I, J). In the prefrontal cortex, BDNF levels were markedly reduced in animals prenatally exposed to poly(I:C), in line with previous evidence showing that MIA induces long-lasting suppression of cortical and hippocampal BDNF, with relevance for synaptic development and affective behaviours [67, 68]. A milder but significant prefrontal reduction was detected after BNT162b2 exposure, without accompanying behavioural impairment, suggesting subtle modulation rather than MIA-like pathology. In the hippocampus, poly(I:C) again decreased BDNF levels, consistent with other models of prenatal neuroinflammation [14, 69]. Neither 3FluArt nor BNT162b2 altered hippocampal BDNF expression. These findings demonstrate that strong maternal immune activation induces long-term neurotrophic alterations, whereas prenatal vaccination does not. The modest prefrontal BDNF reduction observed after BNT162b2 exposure was not accompanied by overt behavioural impairment, indicating that it may reflect subtle modulation rather than disrupted neurodevelopment. Sex-specific analyses of PFC and HP BDNF are provided in S8 Fig.

Altogether, our findings indicate that while strong maternal immune activation can modify fetal neurotrophic signalling, prenatal influenza or COVID-19 vaccination does not alter fetal BDNF levels and therefore does not suggest disrupted neurodevelopment.

TBARS analysis in embryonic brains revealed no significant lipid peroxidation in vaccine-treated groups compared to controls (Fig. 8F), suggesting that maternal vaccination does not induce significant lipid peroxidation in the embryonic nervous system. This result is in line with previous scientific studies that also found no significant change in the marker of lipid peroxidation, TBARS, in either the placenta of pregnant women or in neuronal cells after COVID-19 mRNA vaccination [70]. These results suggest that maternal vaccination does not induce oxidative stress in fetal brains, a key safety consideration.

To further evaluate central innate immune responses, we performed Iba-1 immunohistochemistry to assess microglial cell density in embryonic brains at E13.5. In the dorsal telencephalon, microglial density showed a trend toward increase in the poly(I:C) and 3FluArt groups compared to saline, although these differences did not reach statistical significance (Fig. 9). In contrast, the ganglionic eminence exhibited a significant increase in Iba-1⁺ cell density in both poly(I:C) and 3FluArt groups relative to saline controls. Notably, the COV VAX group did not differ from saline in either region.

These findings are consistent with the well-characterized effects of experimental maternal immune activation. Prenatal exposure to innate immune stimuli such as poly(I:C) can lead to increased microglial proliferation and recruitment in the fetal brain (e.g., [71–73]), reflecting altered neuroimmune development. Microglia are highly dynamic regulators of synaptogenesis, circuit refinement, and developmental apoptosis (see [74, 75]), and region-specific changes in microglial density have been linked to later behavioural phenotypes in rodents following prenatal inflammatory challenges.

The increase in Iba-1⁺ cell density in the ganglionic eminence after 3FluArt exposure suggests that even mild or adjuvant-mediated peripheral immune activation modestly influences microglial distribution in a region-specific manner without evidence of sustained inflammatory programming or accompanying robust cytokine elevation. Similar subtle microglial responses to peripheral immune stimuli have been reported, indicating that microglia can respond to a range of systemic signals without overt pathology (e.g., [76, 77]).

In contrast, maternal BNT162b2 vaccination did not alter Iba-1⁺ density relative to controls and was not associated with fetal CRP induction or BDNF elevation, despite a selective increase in fetal P2X7 receptor levels. Together with the behavioural and cytokine data, these findings indicate the absence of sustained neuroinflammatory programming following maternal vaccination.

Importantly, our analysis quantified total Iba-1⁺ cell density and did not distinguish between morphological activation states; therefore, the observed changes likely reflect altered microglial number or distribution rather than definitive evidence of functional activation.

The P2X7 receptor, involved in inflammasome activation and cytokine release, has been implicated in MIA models [10, 11]. While soluble P2X7 (sP2X7) is considered a circulating marker associated with inflammasome activity, it does not directly reflect receptor activation or membrane localization.

In the present study, maternal plasma sP2X7 levels remained unchanged 24 h after treatment. In addition, we quantified total P2X7 receptor protein levels in embryonic brain tissue and in adult offspring prefrontal cortex and hippocampus. Embryonic P2X7 levels were elevated following poly(I:C) and COV VAX exposure, whereas no persistent alterations were detected in adult brain regions.

ELISA-based quantification reflects total P2X7 protein abundance. It does not distinguish membrane localisation, activation state, ATP sensitivity, pore formation, inflammasome engagement, or cell-type specificity. Therefore, while developmental regulation of P2X7 expression was identified, functional activation or causal involvement in behavioural outcomes cannot be inferred. Future studies assessing receptor localisation and inflammasome components (e.g., caspase-1 activation, IL-1β maturation) are required.

A methodological limitation of the present study is the absence of an irrelevant (non-coding) mRNA–LNP control group. Such a control would help distinguish antigen-specific effects of BNT162b2 from responses to the mRNA platform itself. However, the primary aim of this work was to compare clinically applied maternal vaccinations with a validated MIA model, rather than to investigate generic mRNA exposure. For this purpose, poly(I:C) provided a robust positive control for innate immune activation. Nevertheless, future studies specifically addressing platform-related effects would further refine interpretation of vaccine-specific findings.

## Conclusion and further consideration

This study supports the neurodevelopmental safety of prenatal COVID-19 vaccination in this mouse model. Although mild, age- and sex-dependent behavioural alterations were detected at specific developmental stages, no consistent or persistent ASD-like behavioural phenotype was observed under the tested conditions. Strong maternal and fetal immune responses and multi-domain behavioural alterations were induced by poly(I:C), whereas vaccination was associated with selective and largely transient neuroimmune modulation.

The timing of immunisation may also influence outcomes. Different gestational stages may yield varying results. While our sampling reflects a single timepoint in the immune response, other windows may reveal different effects. Importantly, vaccination did not induce fetal CRP elevation, BDNF upregulation, or persistent P2X7 receptor alterations, and a consistent ASD-relevant behavioural profile was not detected in the behavioural domains assessed.

Further studies are needed to clarify the mechanistic links between maternal immune activation and autism-like behaviour, including the coordinated roles of cytokine signalling, purinergic pathways such as P2X7, and neurotrophic factors in maternal–fetal immune interactions. Translation to humans requires carefully designed clinical studies. These should validate the findings and establish the safety profile of prenatal vaccination across different populations, gestational windows, and developmental trajectories.

## Supplementary information

Supplementary

## Data Availability

Further information and requests for resources should be directed to and will be provided by the Lead Contact, Beata Sperlagh (sperlagh@koki.hu). This paper did not generate new codes.
