# Supplementary material for: Exploring offspring behaviour after prenatal COVID-19 vaccination in mice
Source: Transl Psychiatry. 2026 Jun 15;16:428. doi: 10.1038/s41398-026-04147-7 (PMC13500820; doi:10.1038/s41398-026-04147-7)
Supplement: Supplementary file 1 [file 41398_2026_4147_MOESM1_ESM.docx]

**Supporting Information for**

Exploring offspring behaviour after prenatal COVID-19 vaccination in mice.

Lilla Otrokocsi^1^, Fruzsina Maácz^1, 2^, Pál Tod^1^, Flóra Gölöncsér^1^*_1_, Beáta Sperlágh^1,2^_1_

*Corresponding author

E-mail: [goloncser.flora@koki.hu](mailto:goloncser.flora@koki.hu)

**This file includes:**

Figure S1 to S7

Tables S1 to S2

**S1 Table. Generalized Linear Models (GLZ) to identify sex-specific neurobehavioural changes induced by treatments (sex of offspring × treatment).**

4-week-old offspring

| **Behaviour tests** | **Wald χ^2^ (df: 3)** | **p-value** |
| --- | --- | --- |
| *Open field test* |  |  |
| Distance travelled | 7.00 | 0.0724 |
| Duration in centre | 4.00 | 0.2613 |
| Circling movement | 2.742 | 0.4332 |
| *Novel object recognition test* |  |  |
| NOR index | 1.2132 | 0.7498 |
| Exploration during test | 3.3091 | 0.3463 |
| Number of interactions | 10.10 | 0.0177 |
| *Elevated plus maze test* |  |  |
| Time spent in open arms | 10.361 | 0.0157 |
| Time spent in closed arms | 5.25 | 0.1546 |
| Head dipping | 34.64 | 0.0000001 |
| *Social preference test* |  |  |
| SP index | 0.6201 | 0.8918 |
| Stranger sniffing during test | 3.00 | 0.3916 |
| Number of interactions | 12.457 | 0.0059 |
| *Self-grooming test* |  |  |
| Grooming duration | 2.316 | 0.5093 |
| Grooming frequencies | 1.295 | 0.7302 |
| *Marble burying test* |  |  |
| Number of marbles buried | 8.530 | 0.0362 |

8-week-old offspring

Count data (head dips, grooming frequency, and rotation counts) were analyzed using Generalized Linear Models (GLZs) with a Poisson distribution and log link, including Treatment, Sex, and their interaction as fixed factors. Continuous and percentage data (e.g., exploration time, intruder sniffing, and other behavioral parameters) were analyzed using GLZs with a Gamma distribution and log link, which are appropriate for positive, right-skewed variables. Wald χ² statistics are reported for main and interaction effects. Significance was set at p < 0.05.

| **Behaviour tests** | **Wald χ^2^ (df: 3)** | **p-value** |
| --- | --- | --- |
| *Open field test* |  |  |
| Distance travelled | 9.80 | 0.0205 |
| Duration in centre | 1.34 | 0.7196 |
| Circling movement | 10.067 | 0.0180 |
| *Novel object recognition test* |  |  |
| NOR index | 1.9176 | 0.5896 |
| Exploration during test | 4.4823 | 0.2138 |
| Number of interactions | 23.39 | 0.000034 |
| *Elevated plus maze test* |  |  |
| Time spent in open arms | 1.0354 | 0.7926 |
| Time spent in closed arms | 0.80 | 0.8590 |
| Head dipping | 11.39 | 0.0098 |
| *Social preference test* |  |  |
| SP index | 4.3260 | 0.2283 |
| Stranger sniffing during test | 7.272 | 0.0637 |
| Number of interactions | 7.81 | 0.050047 |
| *Self-grooming test* |  |  |
| Grooming duration | 5.946 | 0.1142 |
| Grooming frequencies | 0.651 | 0.8847 |
| *Marble burying test* |  |  |
| Number of marbles buried | 13.427 | 0.0037 |

**S2 Table. Sex-specific behaviour.**

| Behaviour tests | 4-week-old offspring | | 8-week-old offspring | | |
| --- | --- | --- | --- | --- | --- |
|  | PIC 10 | | | | |
|  | Male | Female | | Male | Female |
| *Open field test* |  |  | |  |  |
| Distance travelled | − | − | | − | ↑↑ |
| Duration in centre | ↓↓↓ | ↓↓ | | − | − |
| Circling movement | − | − | | − | ↑ |
|  |  |  | |  |  |
| *Elevated plus maze test* |  |  | |  |  |
| Time spent in open arms | − | ↑ | | − | − |
| Time spent in closed arms | − | ↓ | | − | − |
| Head dipping | ↓ | − | | − | − |
|  |  |  | |  |  |
| *Social preference test* |  |  | |  |  |
| SP index | ↓ | − | | − | ↓↓ |
| Stranger sniffing during test | ↓ | − | | − | − |
| Number of interactions | − | − | | ↑ | ↑ |
|  |  |  | |  |  |
| *Self-grooming test* |  |  | |  |  |
| Grooming frequencies | ↑ | − | | ↑ | ↑ |
| *Marble burying test* |  |  | |  |  |
| Number of marbles buried | − | − | | ↑↑↑ | − |
|  | COV VAX | | | | |
|  | Male | Female | | Male | Female |
| *Open field test* |  |  | |  |  |
| Duration in centre | ↓↓ | ↓ | | − | − |
|  |  |  | |  |  |
| *Elevated plus maze test* |  |  | |  |  |
| Time spent in closed arms | ↓↓↓ | ↓↓↓ | | − | − |
|  |  |  | |  |  |
| *Self-grooming test* |  |  | |  |  |
| Grooming frequencies | ↑ | − | | − | − |

**S1 Fig. Pooled data of male and female offspring in open field test.**

**S2 Fig. Pooled data of male and female offspring in novel object recognition test.**

**S3 Fig. Pooled data of male and female offspring in elevated plus maze test.**

**S4 Fig. Pooled data of male and female offspring in social preference test.**

**S5. Fig. Pooled data of male and female offspring in self-grooming and marble burying tests.**

**S6 Fig. Gel electrophoresis of BNT162b2 vaccine (right lane).** Left lane, 100-1000 bp DNA ladder.

**S7 Fig Sex-specific P2X7 receptor levels in the PFC and HP of offspring**

HP, hippocampus, P2X7R, P2X7 receptor, PFC, prefrontal cortex

**S8 Fig Sex-specific BDNF levels in the PFC and HP of offspring**

BDNF, brain-derived neurotrophic factor, HP, hippocampus, PFC, prefrontal cortex
